# Behavioral and Other Characteristics Associated with HIV Viral Load in an Outpatient Clinic

**DOI:** 10.1371/journal.pone.0166016

**Published:** 2016-11-02

**Authors:** Paul L. Sacamano, Jason E. Farley

**Affiliations:** 1 Department of Epidemiology, Johns Hopkins University Bloomberg School of Public Health, Baltimore, Maryland, United States of America; 2 Department of Community and Public Health, Johns Hopkins University School of Nursing, Baltimore, Maryland, United States of America; University of Maryland School of Medicine, UNITED STATES

## Abstract

Persons living with HIV (PLWH) who are engaged in care, yet not virally suppressed, represent a risk for transmission and opportunity for risk reduction interventions. This study describes characteristics of an outpatient clinic cohort of PLWH by laboratory confirmed viral suppression status and examines associations with demographics and sexual and drug use behaviors gathered through questionnaire. From a sample of 500 clinic patients, 438 were prescribed antiretroviral treatment (ART) and 62 were not. Among the 438 on ART, 72 (16.4%) were not virally suppressed at the most recent lab draw. Compared to individuals with a suppressed viral load, those that were unsuppressed were more likely to: be black (79.2% vs. 64.2%; *p* = 0.014); earn below $25,000/year (88.9% vs. 65.0%; *p* < 0.001); be of a younger age (47.8 vs. 50.0 mean years; *p* = 0.009); be on opiate substitution (14.1% vs. 6.3%; *p* = 0.023); and acknowledge poly-substance (38.9% vs. 24.4%; *p* = 0.012) and excessive alcohol use (13.9% vs. 6.0%; *p* = 0.019). Conversely, a smaller proportion of those with an unsuppressed viral load had multiple sex partners in the previous 30 days (39.8% vs. 58.5%; *p* = 0.003). In multivariable regression of those on ART, the prevalence of an unsuppressed viral load was 3% lower with each increasing year of age (aPR: 0.97; 95% CI: 0.95, 0.99) and 47% lower with income over $25,000/year (aPR: 0.33; 95% CI: 0.16, 0.70). In a separate analysis of all 500 subjects, ART was less frequently prescribed to blacks compared to whites, heterosexuals, those with lower education and income, and persons with active substance use. Findings confirm that a large proportion of PLWH and engaged in care were not virally suppressed and continued behaviors that risk transmission, indicating the need for screening, prevention counseling and access to ancillary services to lower the incidence of HIV infections.

## Introduction

In the United States, 4% of persons living with HIV (PLWH) diagnosed and retained in care have not been prescribed antiretroviral treatment (ART) and are not virally suppressed, while 10% on ART have not achieved viral suppression.[1] Risky sexual behavior with a person who has an unsuppressed viral load accounts for an estimated 28.9 new infections per 100 persons.[2] Although transmission from injection drug use has declined, substance use is associated with sexual behaviors that risk transmission and may also impair adherence to ART. The purpose of this study is to describe a clinic cohort of PLWH and examine associations with viral suppression with a focus on drug use and sexual risk behaviors. Study findings can inform prevention efforts to reduce risk behaviors among PLWH with unsuppressed viral load seen in the clinical setting.

Substance use is a key factor for high-risk sexual behavior.[3–5] Nationally, 17% of heterosexual women, 16% of men who have sex with men (MSM) and 11% of heterosexual men living with HIV have an unsuppressed viral load concurrent with a sexual or drug use risk behavior.[2] Alcohol and crack or powder cocaine use have been related to multiple concurrent sex partners among heterosexuals and MSM,[6] and heroin use with fewer partners.[7] Inconsistent condom use occurs with alcohol [6, 8, 9] and injection drug use.[10]

Limited access to treatment and low adherence to ART may be a consequence of substance use, but for those who do adhere to treatment virologic outcomes are comparable to those who do not use substances.[11] Use of stimulants, including methamphetamine [12] crack [12, 13] and cocaine [12, 14] as well as alcohol [13,15] but not heroin [14] have been associated with poorer adherence to ART compared to persons who do not use substances. Persons who actively inject drugs have had lower rates of viral suppression at ART initiation but not with continued treatment, possibly due to varying intensity of injection over time, including abstinence, as well as linkages with support services that can improve adherence for those engaged in care.[16]

## Methods

This is a secondary analysis of a cross-sectional sample of 500 PLWH from a study evaluating risk factors for methicillin-resistant *Staphylococcus aureus* colonization.[17] Subject recruitment, interviews and chart abstraction occurred between March 2010 and April 2011. The Johns Hopkins Medicine Institutional Review Board approved the study and consent procedure and all participants provided written informed consent that was recorded on a signed consent form.

### Study population

The Baltimore, Maryland, metropolitan area has the third highest prevalence of PLWH in the US.[18] Similar to national trends, the proportion of incident cases is highest in the MSM risk group (50%), followed by heterosexual (37%) and persons who inject drugs (PWID) (10%).[19]

The Johns Hopkins University AIDS Service (JHUAS) has two clinics in the Baltimore area. Over 75% of JHUAS clients receive care at the Moore Clinic for HIV at the Johns Hopkins Hospital medical campus in the East Baltimore community. The Moore Clinic provides many co-located ancillary services, including opiate substitution, counseling and mental health care, and medication support. The Moore Clinic follows an average of 2,300 mostly African-American (77%) clients annually, including the uninsured with a majority having HIV transmission risks of intravenous drug use and heterosexual sex. The Green Spring Station (GSS) clinic is located in Baltimore County, serves about 650, primarily white (64%) and exclusively insured clients, with MSM as the key affected population.

Eligible participants were current JHUAS clients with two or more visits within the previous 12 months, were 18 years of age and older and able to understand spoken English and provide consent. Clients at each clinic were approached and screened consecutively at a routine or acute care visit unrelated to the study during the recruitment period. Interviewers approached all patients at GSS and every 10^th^ patient entering the Moore Clinic because of its much larger clinic population. A $25 gift card was offered for study participation.

### Behavioral assessment

Study team members administered the questionnaire (S1 Appendix) during face-to-face interviews onsite following clients’ appointments with their providers. Fourteen questions developed from previously published studies [20, 21] focused on characterization of sexual and substance use behaviors. Given the sensitive nature of the information, interviews took place in private consultation rooms and it was stressed that all responses were confidential. Interviewers were registered nurses or nursing students trained in use of the tool as well as sexual history taking. The time frame for any sexual or drug use behaviors was the 12 months prior to the interview. Research nurses reviewed patient medical charts to confirm demographic information and obtain medical history and viral load results from the most recent lab visit. Viral suppression was defined as viral load below detectable limits of 50 HIV-1 RNA copies/mL blood.

### Statistical analysis

The primary analysis examined associations between participant characteristics and behaviors with having an unsuppressed viral load among those prescribed ART. Given the high prevalence of unsuppressed viral load in the cohort, prevalence ratios were determined through a generalized linear model with log link, Poisson distribution and robust standard errors. Patterns of ART prescription were examined comparing persons prescribed and not prescribed ART in the full cohort. Interactions between drug use and sexual behaviors and both risk behaviors with demographic characteristics were tested. Variables with a significance of *p* value of 0.05 or less in bivariate analyses were included in multivariable regression models, except for use of individual substances to avoid collinearity given the high levels of poly-substance use. Condom use was retained regardless of statistical significance given its importance to preventing HIV transmission. All tests were two-sided and a *p* value of 0.05 or less was considered statistically significant. The data analysis was conducted using STATA software version 14 (Stata Corp., College Station, Texas, USA).

## Results

There were 500 participants recruited for the study, 150 from GSS and 350 from the Moore Clinic. There were 438 subjects on ART, of which 366 (83.6%) were virally suppressed and 72 (16.4%) were unsuppressed (Table 1). The median viral load was 8,663 (IQR 250–38,380). Individuals with an unsuppressed viral load were more likely than those with a suppressed viral load to be younger (mean age 47.8 vs. 50.9 years; *p* = 0.009), black compared to other races (79.2% vs. 64.2%; *p* = 0.014) and earn less than $25,000 annually (88.9% vs. 65.0%; *p* < 0.001). Among the unsuppressed, sexual risk was lower, with fewer having multiple sex partners in the previous thirty days (39.8% vs. 58.5%; *p* = 0.003), while substance use was greater, with more using multiple substances (38.9% vs. 24.4%; *p* = 0.012), excessive alcohol (13.9% vs. 6.0%; *p* = 0.019) and benzodiazepines (16.7% vs. 8.5%; *p* = 0.033). Benzodiazepines were the most commonly used substance in combination with others. The unsuppressed were also more likely to be taking opiate substitution therapy (14.1% vs. 6.3%; *p* = 0.023).

**Table 1:** Characteristics by viral suppression among persons prescribed ART.

		Suppressed	Unsuppressed Viral	*p*-value
	Overall N = 438	Viral Load N = 366	Load N = 72	
	N (%)	N (%)	N (%)	
Age, mean years (range)	50.4 (22.7–80.9)	50.9 (25.8–80.9)	47.8 (22.7–63.4)	0.009
Sex				
Female	143 (32.6)	121 (33.1)	22 (30.6)	0.679
Male	295 (67.4)	245 (66.9)	50 (69.4)	
Race				
White/other ^a^	146 (33.3)	131 (35.8)	15 (20.8)	0.014
Black	292 (66.7)	235 (64.2)	57 (79.2)	
Education				
High school or less	229 (52.3)	179 (48.9)	50 (69.4)	0.001
Any college	209 (47.7)	187 (51.1)	22 (30.6)	
Sexual orientation				
Heterosexual	288 (65.7)	235 (64.2)	53 (73.6)	0.124
Gay/lesbian/bisexual	150 (34.3)	131 (35.8)	19 (26.4)	
Annual income				
< $25,000	302 (69.0)	238 (65.0)	64 (88.9)	< 0.001
≥ $25,000	136 (31.0)	128 (35.0)	8 (11.1)	
CD4+ T cell count				
< 200	45 (10.3)	26 (7.1)	19 (26.4)	<0.001
200–349	81 (18.5)	60 (16.4)	21 (29.2)	
350–500	96 (21.9)	76 (20.8)	20 (27.8)	
> 500	216 (49.3)	204 (55.7)	12 (16.7)	
Sexually active				
No	147 (35.6)	124 (33.9)	23 (31.9)	0.751
Yes	291 (66.4)	242 (66.1)	49 (68.1)	
Condom use				
Inconsistent/never	152 (52.2)	125 (51.7)	27 (55.1)	0.659
Always	139 (47.8)	117 (48.3)	22 (44.9)	
>1 sex partner ^b^				
No	275 (46.4)	103 (41.5)	53 (60.2)	0.003
Yes	29 (53.6)	145 (58.5)	35 (39.8)	
Poly-substance use ^c^				
No	319 (73.2)	275 (75.6)	44 (61.1)	0.012
Yes	117 (26.8)	89 (24.4)	28 (38.9)	
Excessive alcohol ^d^				
No	405 (92.7)	343 (94.0)	62 (86.1)	0.019
Yes	32 (7.3)	22 (6.0)	10 (13.9)	
IV heroin and/or cocaine				
No	421 (96.1)	353 (96.5)	68 (94.4)	0.421
Yes	17 (3.9)	13 (3.5)	4 (5.6)	
Crack cocaine				
No	361 (82.6)	303 (83.0)	58 (80.6)	0.615
Yes	76 (17.4)	62 (17.0)	14 (19.4)	
Benzodiazepines				
No	394 (90.2)	334 (91.5)	60 (83.3)	0.033
Yes	43 (9.8)	31 (8.5)	12 (16.7)	
Opiate substitution				
No	404 (92.5)	343 (93.7)	61 (85.9)	0.023
Yes	33 (7.5)	23 (6.3)	10 (14.1)	

Suppressed below 40 copies HIV-1 RNA particles from most recent laboratory results within the prior 12 months.

^a^ Latino/Hispanic: 8 (1.6%); Asian/Pacific Islander: 2 (0.4%); Native American: 3 (0.6%).

^b^ Previous 30 days.

^c^ Any marijuana; crack cocaine; prescription pain medication; benzodiazepines; excessive alcohol; intranasal heroin or cocaine; IDU heroin or cocaine; ecstasy; smoked heroin; crystal (methamphetamine); poppers over prior 12 months.

^d^ Excessive alcohol use was self-defined by the participant.

Associations modeled with viral suppression are given for those taking ART in Table 2. In univariate regression, lower prevalence of an unsuppressed viral load was associated with each year of increasing age (PR: 0.97; 95% CI: 0.95, 0.99), any college (PR: 0.48; 95% CI: 0.30, 0.77), annual income equal to or over $25,000/year (PR: 0.28; 95% CI: 0.14, 0.56) and with use of benzodiazepines (PR: 0.55; 95% CI: 0.32, 0.93). Greater prevalence of unsuppressed viral load was associated with black race (PR: 1.9; 95% CI: 1.11, 3.24), poly-substance use (PR: 1.74; 95% CI: 1.14, 2.65) and opiate substitution (2.01; 95% CI: 1.14, 3.54). In multivariable regression, only the lower prevalence of being unsuppressed with increasing age (aPR: 0.97; 95% CI: 0.95, 0.99) and income equal to or over $25,000/year (aPR: 0.33; 95% CI: 0.16, 0.70) remained significant.

**Table 2:** Univariate and multivariable regression results for the outcome of having an unsuppressed viral load for those taking ART.

Variable	Univariate regression		Multivariable regression	
	PR (95% CI)	*p*-value	PR ratio (95% CI)	*p*-value
Sexual orientation				
Gay/lesbian/bisexual	1.00	0.132		
Heterosexual	1.45 (0.89, 2.36)			
Age (years)	**0.97 (0.95, 0.99)**	0.010	**0.97 (0.95, 0.99)**	0.009
Sex				
Female	1.00			
Male	0.91 (0.57, 1.44)	0.680		
Race				
White/other	1.00	0.018	1.00	0.228
Black	**1.9 (1.11, 3.24)**		1.40 (0.81, 2.41)	
Education				
High school or less	1.00	0.002		
Any college	**0.48 (0.30, 0.77)**			
Income				
< $25,000/year	1.00	<0.001	1.00	0.004
≥ $25,000/year	**0.28 (0.14, 0.56)**		**0.33 (0.16, 0.70)**	
Sexually active ^a^	0.93 (0.59, 1.46)	0.751		
>1 sex partner ^b^	0.75 (0.29, 1.92)	0.543		
Condom use				
Always	1.00	0.445	1.00	0.356
Inconsistent	0.85 (0.56, 1.29)		0.82 (0.54, 1.24)	
Poly-substance use	**1.74 (1.14, 2.65)**	0.011	1.18 (0.75, 1.88)	0.470
Injection drugs ^c^	1.46 (0.60, 3.53)	0.405		
Excessive alcohol ^d^	**0.49 (0.28, 0.86)**	0.013	1.21 (0.67, 2.19)	0.528
Opiate substitution	**2.01 (1.14, 3.54)**	0.016		
Benzodiazepines	**0.55 (0.32, 0.93)**	0.026		
Crack cocaine	0.87 (0.51, 1.48)	0.613		

CI, confidence interval; PR, prevalence ratio

Note: Bold text denotes statistically significant findings at *p* ≤ 0.05.

^a^ Previous 12 months.

^b^ Previous 30 days.

^c^ Heroin, cocaine or both.

^d^ Excessive alcohol use was self-defined by the participant.

Among the full cohort of 500 participants, 62 were not prescribed ART. Patterns of ART prescription revealed greater proportions prescribed ART with any college compared to high school education (92.5% vs. 83.6%; *p* = 0.003) and with incomes over $25,000/year (92.5% vs. 85.6%; *p* = 0.031). Prescription of ART was lower among blacks compared to other races (84.6% vs. 94.2%; *p* = 0.003), persons who use multiple substances (81.3% vs. 90.1%; *p* = 0.007), injection drugs (65.4% vs. 88.8%; p < 0.001), excessive alcohol (74.4% vs. 88.8%; *p* = 0.006) and crack cocaine (78.4% vs. 89.8%; *p* = 0.002), as well as among heterosexuals compared to gay/lesbian/bisexuals (85.2% vs. 92.6%; *p* = 0.019). The differences between sexual orientation may be related to socioeconomic characteristics, with persons identifying as gay/lesbian/bisexual more likely to have some college education (75.9% vs. 30.5%; *p* < 0.001) and earn more than $25,000 annually (58.6% vs. 15.4%; *p* < 0.001).

## Discussion

While sex and drug use behaviors were not associated with an unsuppressed viral load, they did occur in the presence of viremia and consequently represent a risk for transmission. Compared to a nationally representative survey of PLWH receiving outpatient care in the United States,[22] the study population had greater transmission risk behaviors, with more reporting unprotected sex (37.8% vs. 23%), crack cocaine use (38.3% vs. 4.3%), and any non-injection drug (37.6% vs. 26.6%) or intravenous drug use (10.2% vs. 2%). Our study also had fewer with a high school or higher education (45.2% vs. 53.2%) and a greater proportion of black race (69% vs. 41.7%), indicating a more socioeconomically disadvantaged group.

Guidelines to initiate ART in treatment naïve patients at the time of the study recommended starting treatment when CD4+ T cell counts declined below 500 cells/mm3 or there was a history of AIDS.[23] In practice, many JHUAS providers were offering ART to all patients, even those with CD4+ T cell levels over 500, and some patients not on ART during the study period had been previously, but did not continue due to low medication adherence. Prior treatment adherence and provider prescribing preferences were not available and consequently it was not possible to distinguish how these factors may have impacted the decision not to treat.

It was also found that ART was less likely to be prescribed to persons of black race, having high school or less education and reporting substance use. Abstinence or participation in mental health and substance use treatment services supports adherence [24] and indicates the importance of screening for psychosocial needs and referral to ancillary care. Although substance use was common in this cohort, it was not found to be associated with viral suppression in the adjusted model, suggesting that patients using substances can be adherent and bias against offering treatment due to substance use behaviors should be avoided. With younger age and lower income related to an unsuppressed viral load, adherence counseling should be culturally sensitive and educationally appropriate for youth, and barriers to accessing care such as transportation needs and unstable housing should be addressed.

Although just 40% of the 1.2 million PLWH in the U.S. are engaged in care,[1] they represent a substantial opportunity to implement risk reduction interventions given that many are not virally suppressed.[22] Interventions to change drug use and sexual risk behaviors can produce significant and lasting reductions in risk behaviors and improve ART adherence.[25–27] Yet among PLWH receiving regular care, prevention counseling to reduce transmission from sexual and substance use behaviors is provided half or less of the time.[28, 29] With high proportions engaging in high-risk drug use and sexual behaviors, our study confirms the need for these services for PLWH seen in the clinical setting. However, risk behavior(s) or viremia alone are necessary but not individually sufficient for transmission to occur, and consequently must be considered together when evaluating transmission risks.[2, 30]

While many HIV specialty clinics provide co-located mental health, drug treatment and other supportive services,[31] low rates of screening, counseling and referrals by clinicians remains a challenge.[28,29] Screening for risk behaviors and providing prevention messages are limited by time constraints and competing clinical priorities even where these interventions are the standard of care for a clinical practice. Additionally, with healthcare reform and the move away from specialty HIV to general primary care clinics, patients face new challenges in navigating the healthcare system to connect with ancillary services.[32]

This study has described the characteristics of clients with an unsuppressed viral load and considered patterns of ART prescription. Limitations included the use of self-reported risk behaviors that are subject to social desirability and recall bias. Important factors to consider in future studies include length of time since diagnosis, stage of HIV disease at diagnosis, duration of ART, and HIV or treatment status of stable partners, which were not determined given the degree of missing data for these particular characteristics and inability to confirm reliability of the information that was available.

## Conclusion

A proportion of persons in HIV medical care on ART were not virally suppressed and continued to participate in transmission risk behaviors, indicating the need for screening, prevention counseling and access to ancillary services, particularly for substance use and safer sex counseling. Substance use itself was not a risk factor for unsuppressed viral load, indicating persons actively using can be adherent to treatment.

## Supporting Information

S1 Appendix Survey questionnaire instrument from the primary study. (DOC)

## References

[1] Bradley H, Hall HI, Wolitski RJ, Van Handel MM, Stone AE, LaFlam M, et al Vital Signs: HIV Diagnosis, Care, and Treatment Among Persons Living with HIV—United States, 2011. MMWR Morb Mortal Wkly Rep 2014 11 28;63(47):1113–1117. DOI mm6347a5 [pii] . 25426654 PMC5779517

[2] Hall HI, Holtgrave DR, Tang T, Rhodes P. HIV transmission in the United States: considerations of viral load, risk behavior, and health disparities. AIDS Behav 2013 6;17(5):1632–1636. 10.1007/s10461-013-0426-z . 23456577

[3] McCarty-Caplan D, Jantz I, Swartz J. MSM and drug use: A latent class analysis of drug use and related sexual risk behaviors. AIDS Behav 2014 7;18(7):1339–1351. 10.1007/s10461-013-0622-x 24065437

[4] Seth P, Wingood GM, DiClemente RJ, Robinson LS. Alcohol use as a marker for risky sexual behaviors and biologically confirmed sexually transmitted infections among young adult African-American women. Womens Health Issues 2011 Mar-Apr;21(2):130–135. 10.1016/j.whi.2010.10.005 21276736 PMC4232951

[5] Purcell DW, Mizuno Y, Metsch LR, Garfein R, Tobin K, Knight K, et al Unprotected sexual behavior among heterosexual HIV-positive injection drug using men: associations by partner type and partner serostatus. J Urban Health 2006 7;83(4):656–668. DOI: 0.1007/s11524-006-9066-1 10.1007/s11524-006-9066-1 16736116 PMC2430482

[6] Newville H, Haller DL. Psychopathology and transmission risk behaviors in patients with HIV/AIDS. AIDS Care 2010 10;22(10):1259–1268. 10.1080/09540121003615111 . 20640950

[7] Maranda MJ, Han C, Rainone GA. Crack cocaine and sex. J Psychoactive Drugs 2004 9;36(3):315–322. 10.1080/02791072.2004.10400032 . 15559679

[8] Hasse B, Ledergerber B, Hirschel B, Vernazza P, Glass TR, Jeannin A, et al Frequency and determinants of unprotected sex among HIV-infected persons: the Swiss HIV cohort study. Clin Infect Dis 2010 12 1;51(11):1314–1322. 10.1086/656809 10.1086/656809 21034200

[9] Vosburgh HW, Mansergh G, Sullivan PS, Purcell DW. A review of the literature on event-level substance use and sexual risk behavior among men who have sex with men. AIDS Behav 2012 8;16(6):1394–1410. 10.1007/s10461-011-0131-8 . 22323004

[10] Celentano DD, Latimore AD, Mehta SH. Variations in sexual risks in drug users: emerging themes in a behavioral context. Curr HIV/AIDS Rep 2008 11;5(4):212–218. PMCID: PMC2801160. 18838061 10.1007/s11904-008-0030-4 PMC2801160

[11] Celentano DD, Lucas G. Optimizing treatment outcomes in HIV-infected patients with substance abuse issues. Clin Infect Dis 2007 12 15;45 Suppl 4:S318–23. 10.1086/522557 . 18190306

[12] Hinkin CH, Barclay TR, Castellon SA, Levine AJ, Durvasula RS, Marion SD, et al Drug use and medication adherence among HIV-1 infected individuals. AIDS Behav 2007 3;11(2):185–194. 10.1007/s10461-006-9152-0 . 16897351 PMC2867605

[13] Mellins CA, Havens JF, McDonnell C, Lichtenstein C, Uldall K, Chesney M, et al Adherence to antiretroviral medications and medical care in HIV-infected adults diagnosed with mental and substance abuse disorders. AIDS Care 2009;21(2):168–177. 10.1080/09540120802001705 . 19229685 PMC5584780

[14] Arnsten JH, Demas PA, Grant RW, Gourevitch MN, Farzadegan H, Howard AA, et al Impact of active drug use on antiretroviral therapy adherence and viral suppression in HIV-infected drug users. J Gen Intern Med 2002 5;17(5):377–381. DOI: jgi10644 [pii] 10.1046/j.1525-1497.2002.10644.x 12047736 PMC1495042

[15] Kalichman SC, Grebler T, Amaral CM, McNerney M, White D, Kalichman MO, et al Viral Suppression and Antiretroviral Medication Adherence Among Alcohol Using HIV Positive Adults. Int J Behav Med 2014;21(5):811–820. 10.1007/s12529-013-9353-7 PMCID: PMC4283596. 24085706 PMC4283596

[16] Kerr T, Marshall BD, Milloy MJ, Zhang R, Guillemi S, Montaner JS, et al Patterns of heroin and cocaine injection and plasma HIV-1 RNA suppression among a long-term cohort of injection drug users. Drug Alcohol Depend 2012 7 1;124(1–2):108–112. 10.1016/j.drugalcdep.2011.12.019 PMCID: PMC3342432. 22245312 PMC3342432

[17] Farley JE, Hayat MJ, Sacamano PL, Ross T, Carroll K. Prevalence and risk factors for methicillin-resistant Staphylococcus aureus in an HIV-positive cohort. Am J Infect Control 2015 4 1;43(4):329–335. 10.1016/j.ajic.2014.12.024 PMCID: PMC4386874. 25687358 PMC4386874

[18] Centers for Disease Control and Prevention. HIV Surveillance Report, 2011. 2013;23. From: http://www.cdc.gov/hiv/pdf/statistics_2011_hiv_surveillance_report_vol_23.pdf

[19] Maryland Department of Health and,Mental Hygiene. HIV in Baltimore City: an epidemiological profile. 2012. From: http://health.baltimorecity.gov/hivstd-data-resources.

[20] Farley JE, Ross T, Stamper P, Baucom S, Larson E, Carroll KC. Prevalence, risk factors, and molecular epidemiology of methicillin-resistant Staphylococcus aureus among newly arrested men in Baltimore, Maryland. Am J Infect Control 2008;36(9):644–650. 10.1016/j.ajic.2008.05.005 PMCID: PMC2603277. 18834755 PMC2603277

[21] Farley JE, Ross T, Krall J, Hayat M, Caston-Gaa A, Perl T, et al Prevalence, risk factors, and molecular epidemiology of methicillin-resistant Staphylococcus aureus nasal and axillary colonization among psychiatric patients on admission to an academic medical center. Am J Infect Control 2013 Mar;41(3):199–203. 10.1016/j.ajic.2012.03.028 . 22999771

[22] Centers for Disease Control and Prevention. Behavioral and Clinical Characteristics of Persons Receiving Medical Care for HIV Infection—Medical Monitoring Project, United States, 2010. HIV Surveillance Special Report 9. From: http://www.cdc.gov/hiv/library/reports/surveillance/#special.

[23] Panel on Antiretroviral Guidelines for Adults,and Adolescents. Guidelines for the use of antiretroviral agents in HIV-1-infected adults and adolescents. 2011. From: http://www.aidsinfo.nih.gov/ContentFiles/AdultandAdolescentGL.pdf.

[24] Malta M, Strathdee SA, Magnanini MM, Bastos FI. Adherence to antiretroviral therapy for human immunodeficiency virus/acquired immune deficiency syndrome among drug users: a systematic review. Addiction 2008 8;103(8):1242–1257. 10.1111/j.1360-0443.2008.02269.x . 18855813

[25] Crepaz N, Lyles CM, Wolitski RJ, Passin WF, Rama SM, Herbst JH, et al Do prevention interventions reduce HIV risk behaviours among people living with HIV? A meta-analytic review of controlled trials. AIDS 2006 1 9;20(2):143–157. 10.1097/01.aids.0000196166.48518.a0 . 16511407

[26] Kalichman SC, Cherry C, Kalichman MO, Amaral CM, White D, Pope H, et al Integrated behavioral intervention to improve HIV/AIDS treatment adherence and reduce HIV transmission. Am J Public Health 2011 3;101(3):531–538. 10.2105/AJPH.2010.197608 PMCID: PMC3036694. 21233431 PMC3036694

[27] Healthy Living Project Team. Effects of a behavioral intervention to reduce risk of transmission among people living with HIV: the healthy living project randomized controlled study. J Acquir Immune Defic Syndr 2007 2 1;44(2):213–221. 10.1097/QAI.0b013e31802c0cae . 17146375

[28] Flickinger TE, Berry S, Korthuis PT, Saha S, Laws MB, Sharp V, et al Counseling to reduce high-risk sexual behavior in HIV care: a multi-center, direct observation study. AIDS Patient Care STDS 2013 7;27(7):416–424. 10.1089/apc.2012.0426 PMCID: PMC3704109. 23802144 PMC3704109

[29] Mizuno Y, Zhu J, Crepaz N, Beer L, Purcell DW, Johnson CH, et al Receipt of HIV/STD prevention counseling by HIV-infected adults receiving medical care in the United States. AIDS 2014 1 28;28(3):407–415. 10.1097/QAD.0000000000000057 PMCID: PMC4645275. 24056066 PMC4645275

[30] Centers for Disease Control and Prevention, Health Resources and SA, National Institutes oH, American Academy of HM, Association of Nurses in,AIDS Care, International Association of Providers of,AIDS Care, et al. Recommendations for HIV Prevention with Adults and Adolescents with HIV in the United States, 2014. 2014. From: https://stacks.cdc.gov/view/cdc/26062.

[31] Ojikutu B, Holman J, Kunches L, Landers S, Perlmutter D, Ward M, et al Interdisciplinary HIV care in a changing healthcare environment in the USA. AIDS Care 2014;26(6):731–735. 10.1080/09540121.2013.855299 . 24191727

[32] Hazelton PT, Steward WT, Collins SP, Gaffney S, Morin SF, Arnold EA. California's "Bridge to Reform": identifying challenges and defining strategies for providers and policymakers implementing the Affordable Care Act in low-income HIV/AIDS care and treatment settings. PLoS One 2014;9(3):e90306 10.1371/journal.pone.0090306 PMCID: PMC3943953. 24599337 PMC3943953

