# Supplementary material for: Behavioral and Other Characteristics Associated with HIV Viral Load in an Outpatient Clinic
Source: PLoS One. 2016 Nov 2;11(11):e0166016. doi: 10.1371/journal.pone.0166016 (PMC5091742; doi:10.1371/journal.pone.0166016)
Supplement: S1 Appendix — (DOC) [file pone.0166016.s001.doc]

**A Methicillin-resistant Staphylococcus aureus (MRSA) Questionnaire**

**Admit Date** ___ ___ / ___ ___ / ___ ___

M M D D Y Y

**Admit Culture Date** ___ ___ / ___ ___ / ___ ___

M M D D Y Y

**Interview Date** ___ ___ / ___ ___ / ___ ___

M M D D Y Y

**Time Started** ___:___ AM PM

HR:MN

**Subject Number: ___________________ Culture Number: ___________________**

**Interviewer Name: ___________________** **Location:** ___________________

#### Before I begin, I would like to tell you a little about this survey. I am going to ask questions about your background, health history, and risk of exposure to the MRSA bacteria. All information is confidential and WILL NOT be shared with others. Your name is not included in this survey. When we have completed the survey, you will also be given time to ask questions of me.

#### Demographic INFORMATION:

Instructions: Please circle the most appropriate answer about your background.

**To begin the interview, I am going to ask you some questions about your background.**

**1. What is your Date of Birth?** ________________

**2. What is your gender?**

1. Male
2. Female

**Race:**

**3. Do you consider yourself to be?**

1. Black / African-American or Black / African descent
2. Latino / Hispanic
3. Caucasian / White
4. Asian / Pacific Islander
5. Native American/American Indian
6. Other (specify) ________________
7. Don’t Know/Refuse

**Ethnicity**:

**4. Do you also consider yourself to be Latino(a)?**

1. Yes
2. No

**Living Situation:**

**5. Are you homeless?**

1. Yes
2. No

**IF YES TO #5, SKIP TO QUESTION # 9**

**6. If no, do you live alone?**

1. Yes
2. No

**IF YES TO #6, SKIP TO QUESTION # 9**

**7. If no, do you live:**

1. with partner, husband/wife
2. with family (more than one other person)
3. in a group home
4. in a nursing home
5. Other (specify) ________________

**8. How many people live in your home (count all household members including yourself)?** __________

**Zip Code:**

**9. What was your primary zip code?** ____ ____ ____ ____ ____

**EDUCATION/INCOME/EMPLOYMENT:**

Instructions: Please circle the most appropriate answer about your education/income questions.

**Education/Income:**

**10. How far did you go in school?**

1. No high school diploma / No GED
2. High school / GED
3. Some college /vocational school
4. College graduate or above

**11. What is your primary source of income?**

1. Work Full-Time
2. Work Part-Time
3. Social Security
4. Disability Income
5. Pension/Retirement
6. Other: _______________________

**12. Is your total estimated yearly income from ALL sources?**

1. < 25,000
2. 25,001 – 50,000
3. 50,001 – 75,000
4. >75,000
5. **Does your job involve hands on contact with customers?**
6. Yes
7. No

**INCARCERATION HISTORY:**

Instructions: In this part of the survey, I will ask you some questions about your health history.

**14. Has anyone in your household, including yourself, been arrested or incarcerated in the last 12 months (such as friend, family member)?**

1. Yes
2. No

**15. In the last 12 months, how many times has another household member (besides yourself) been arrested or incarcerated?**

1. None
2. Once
3. 2 – 3
4. 4 or more

**16. In the last 12 months, how many times have you been arrested or incarcerated?**

1. None
2. Once
3. 2 – 3
4. 4 or more

**IF NONE TO #16, SKIP TO QUESTION # 25**

**17. How long was your most recent incarceration/arrest? _____________**

**18. Where were you locked up? _______________________**

**19. During your previous incarceration/arrest, did you develop a skin infection, abscess, spider bite?**

1. Yes
2. No

**20. During your previous incarceration/arrest, did you have a cell mate with a skin infection, abscess, or spider bite?**

1. Yes
2. No

**21. During your previous incarceration/arrest, did you use a weight room/work out facility?**

1. Yes
2. No

**22. During your previous incarceration/arrest, did you have a work assignment/job?**

1. Yes - _________________________________
2. No

**23. During your previous incarceration/arrest, did you have a sick call visit for any reason?**

1. Yes - _________________________________
2. No

**24. During your previous incarceration/arrest, did you shower daily?**

1. Yes
2. No

**HEALTH HISTORY:**

Instructions: In this part of the survey, I will ask you some questions about your health history.

**25. During the last 12 months, how would you describe your health outside of the reason you are here?**

1. Excellent
2. Good
3. Fair
4. Poor

**26. What year were you diagnosed with HIV? _________**

**27. Do you current take HIV medications?**

1. Yes
2. No

**28. In the last 12 months, have you been hospitalized for medical reasons?**

If **Yes,** for what reason? _________________________

**29. During any previous hospitalization were you on isolation for any reason?**

**(**Meaning did the nurse or doctor wear gowns and gloves when speaking with you in your room?)

1. Yes - why? ___________________________
2. No

**30. Have you ever been tested for MRSA (this would be by placing a cotton swab in your nose)?**

1. Yes
2. No

**31. If yes, was this during a hospital admission or as an outpatient visiting the clinic?**

1. Hospital admission
2. Outpatient visit

**32. In the last 12 months, have you taken any pills for treatment of an infection (antibiotics)?**

1. Yes – where was the infection _____________________________
2. No

**IF NO TO QUESTION 32, SKIP TO # 34**

**33. When did you stop taking the antibiotic?**

1. When I felt better
2. When I ran finished the prescription

**34. During the last 12 months, have any of your household members been admitted to a hospital for more than 2 days?**

1. Yes
2. No

**35. In the past 12 months, have you routinely played team sports (such as football, wrestling, basketball)?**

1. Yes
2. No

**36. In the last 12 months, did you routinely visit a gym?**

1. Yes
2. No

**37. Have you been diagnosed with an STD in the last 6 months?**

1. Yes
2. No

**38. If so, which one?**

1. Chlamydia
2. Gonorrhea
3. Syphilis
4. Human papillomavirus (genital warts)
5. Trichomoniasis
6. Bacterial vaginosis (BV)
7. Genital herpes

(Note: Pictures of skin abscess should be shown to participant for the following questions)

**39. Do you currently have an abscess, boil, spider bite or skin infection?**

1. Yes

2. No

**40. Have you had an abscess, boil, spider bite or skin infection in the last 12 months?**

1. Yes

2. No

**IF NO, TO 39 AND 40 SKIP TO QUESTION 51**

**41. How many times in the last 12 months have you had a skin abscess, boil, spider bite or skin infection?** (estimate)

1. 1 – 2
2. 3 – 4
3. More than 4

**42. What was the date when you last had an abscess, boil, spider bite, or skin Infection? ___ ___ /___ _**

MM / YY

**43. Where was your abscess, boil, spider bite or skin infection treated?**

1. Nowhere, I treated it myself (Skip to Question # 39)
2. Urgent Care Center (Patient First, etc)
3. Emergency Department (including Urgent Care Centers within ED’s)
4. Primary Care Clinic (Doctor’s Office)
5. Other: ________________________

**44. Did you get any antibiotics for your abscess, boil, spider bite, or skin infection?**

1. Yes, **Do you know the name of the antibiotic** (**specify**)**:_________________**

2. No

**45. Did you finish all of the antibiotics you were given for this purpose?**

1. Yes

2. No

**46. If you treated the boil/abscess/spider bite yourself, did you use any antibiotics given to you by friends or family from “left over” antibiotics?**

1. Prescribed

2. Friends/Family

**47. Did you require hospitalization, even over night, for your abscess, boil, spider bites or skin infection?**

1. Yes

2. No

**48. If so, how many days were you hospitalized for your abscess, boil, spider bite or skin infection?**

1. 1 – 2 days
2. greater than 2 days, but less than 7 days
3. greater than 7 days

**48. Have any of your household members had an abscess, boil, spider bite, or skin infection?**

1. yes

2. no

3. unknown

**50. You said you have ____ household members (see question # 7 above). How many of these household members have had an abscess, boil, spider bite or skin infection in the last year?** _______

#### BEHAVIORAL RISK FACTORS:

Instructions: The next set of questions asks about your personal behaviors: Please remember that this information is not shared with correctional officers or law enforcement unless you state a desire to harm yourself or another person.

**51. In the last 12 months, have you had sex (oral, anal or other physical contact leading to at least one partner ‘getting off’ or having an orgasm)?**

1. Yes – Do you have a primary (main) sexual partner that you have sex with? YES NO
2. No

**52. Was this with?**

1. Women
2. Men
3. Both sexes

**IF ANSWER IS WOMEN (#1) TO QUESTION 52, SKIP TO QUESTION 54**

**53. If you have sex with men, during sex are you primarily?**

1. Top
2. Bottom
3. Versatile (50/50)
4. I do not have anal sex

**54. In the last 12 months, have your sexual practices ever included: (Select all that apply)**

1. Giving oral sex (mouth to any sex organs)
2. Receiving oral sex
3. Giving oral-anal sex (riming)
4. Receiving oral-anal sex (riming)
5. Vaginal sex
6. Giving anal sex
7. Receiving anal sex
8. More than one partner per encounter
9. Fisting
10. Sex toys
11. Leather harnesses, slings, whips or similar objects during sex

**55. What percentage of the time do you use condoms during sex (each sex activity equals one sex act – i.e. a patient who reports a sexual encounter with 1 person, but having oral and anal sex would have 2 sex acts)?**

1. Never
2. 25% (1 in every 4 sex acts)
3. 50% (1 in every 2 sex acts)
4. 100% (everytime including oral sex)

**56. How many sexual partners would you say you have had over your lifetime? ____________**

**57. Are you currently in a monogamous relationship for the last 12 months?**

1. Yes
2. No

**58. How many sexual partners would you say you have had In the last year? _____________**

**59. In the last 30 days? ____________**

**60. In the last 12 months, have you had sex with a person with a boil, spider bite or skin infection anywhere on their body?**

1. Yes
2. No

**61. In the last 12 months, has anyone given you money, drugs or anything of value (such as food or a place to stay) to have sex?**

1. Yes
2. No

**62. In the last 12 months, have you given anyone money, drugs or anything of value (such as food or a place to stay) to have sex?**

1. Yes
2. No

**63. In the last 12 months, have you used any street drugs (such as heroin, cocaine, crystal meth)?**

1. Yes
2. No

**64. Which drugs do you currently use or have used in the last 12 months? Select all that apply**

1. IV Heroin
2. Intranasal (snort) Heroin
3. Smoked Heroin
4. Heroin and Tylenol (Cheese)
5. IV Cocaine
6. Intranasal (snort) cocaine
7. Smoked Cocaine
8. Crystal Methamphetamine (Tina, Crank, Crystal, Speed)
9. Ecstasy or “E”
10. Prescription pain killers (oxycodone, OxyContin, Percocet, Dilaudid, Vicodin)
11. Benzodiazepines (Ativan, Xanax, Valium, Klonopin)
12. Marijuana
13. Methadone
14. Bupernex / Subutx / Naltrexon and Suboxone
15. Seroquel (Quell)
16. Clonidine
17. Nitrates (“poppers”)
18. ED Medications (Viagra; Cialis; Levitra)
19. Excessive alcohol
20. Other _______________________________

**That was my last question. Now that the survey has ended, would you like to go back and make changes to any of your previous responses? (if yes, mark change next to original answer above). Thank you for participating in this study and for your patience. Do you have any questions for me?**

**Time Finished** ___:___ AM PM

HR:MN

**TO BE COMPLETED BY RA REVIEW OF THE JOHNS HOPKINS ELECTRONIC PATIENT RECORD:**

**Medical Record Screening Date** ___ ___ / ___ ___ / ___ ___

M M D D Y Y

**What was the most recent T-cell count? ____________ CD4 % _____________**

**What is the lowest CD4 count (Nadir)? _______________**

**What was the most recent Viral Load? ______________**

**Is the patient on HIV medications?**

1. Yes
2. No

**List HIV medications:**

**________________________________ ________________________________**

________________________________ **________________________________**

________________________________ **________________________________**

**Any medications for infection prevention (such as Bactrim or Azithromycin)?**

1. Yes
2. No

**Which prophylactic medications?**

1. trimethoprim/sulfamethoxezole (Bactrim)
2. dapsone (Aczone)
3. azithromycin (Zithromax)
4. fluconazole (Diflucan)

**Toxicology Screen Available?**

1. Yes For what substance(s)? _________________________
2. No

**What is/are the current medical diagnosis in addition to HIV?**

1. **________________________________ 2. ________________________________**

**Has the subject previously been tested for MRSA?**

1. Yes
2. No

**If yes, was the subject positive?**

1. Yes
2. No

**If yes, what was the culture for surveillance or clinical reasons?**

1. Surveillance (nares only)
2. Clinical
3. Both

**What was the date of the most recent positive MRSA culture?** ___ ___ / ___ ___ / ___ ___

M M D D Y Y

**FOR INVESTIGATOR USE ONLY:**

**Subject Number: ___________________ Specimen Number: ___________________**

**Date Collected:** **___________________ Date Resulted:** **___________________**

**FINAL MICROBIOLOGY DATA VERIFICATION FORM:**

**Attach all final culture and susceptibility results**

**MRSA: MLST typing Result:** _____________________

1. Positive (insert strain type number)
2. Negative
3. Unknown

**Positive Sites (select all that apply):**

- Nares (both nostrils)
- Throat (posterior pharynx)
- Axillae (both axillae)
- Groin (inguinal area)
- Perineum (between scrotum and anus; between vagina and anus)
- Vaginal (moisten swab)
- Rectal (moisten swab)
- Wound culture obtained (if applicable)

**SCCmec Typing**

1. I
2. II
3. III
4. IV a/b/c/d
5. V
6. VI

**PVL:**

1. Positive
2. Negative
3. Indeterminate

**TSST1:**

1. Positive
2. Negative
3. Indeterminate

**Mupiricin Resistant:**

1. Sensitive
2. Resistant

**Phoenix Antimicrobial Susceptibilities Completed:**

1. Yes
2. No
